# Vision Loss in Late Life: An Overview

**DOI:** 10.1093/geroni/igaf122.1739

**Published:** 2025-12-31

**Authors:** Brooke Donaher

**Affiliations:** University of Rochester, Rochester, New York, United States

## Abstract

Vision loss is not synonymous with aging, but the risk for vision impairment increases with age. By 2050 there will be 1.28 billion older people with avoidable vision impairment (De la Fuente-Núñez et al ., 2023). The purpose of this presentation is to provide an overview of the medical and ophthalmological issues that contribute to vision loss among older adults. The most common causes of vision loss are refractive error and diseases associated with aging (but not necessarily caused by aging), namely cataracts, glaucoma, age-related macular degeneration (the leading cause of blindness in those over 60), and diabetic retinopathy (leading cause of blindness in those 20-74). Refractive error and cataracts can be treated and fully corrected in most cases. Vision loss from glaucoma, macular degeneration and diabetic eye disease can be prevented when identified and treated early. However, these age-related eye diseases cannot be cured and a late diagnosis can result in permanent vision loss. Macular degeneration and diabetic retinopathy are associated with central vision loss or blind spots in the visual field, whereas glaucoma causes loss of peripheral vision. Vision and eye health screenings and comprehensive eye exams for older individuals are recommended every one to two years to reduce the risk for permanent vision loss. Such preventive measures contribute to healthy aging. De la Fuente-Núñez V, Bouzanis K, Joseph S. Connecting healthy ageing and vision: Report summary; 2023.

